# Serum Gamma-Glutamyltransferase, Daily Alcohol Consumption, and the Risk of Chronic Kidney Disease: The Kansai Healthcare Study

**DOI:** 10.2188/jea.JE20180240

**Published:** 2020-04-05

**Authors:** Mikiko Shibata, Kyoko Kogawa Sato, Shinichiro Uehara, Hideo Koh, Keiko Oue, Hiroshi Kambe, Michio Morimoto, Tomoshige Hayashi

**Affiliations:** 1 Preventive Medicine and Environmental Health, Osaka City University Graduate School of Medicine, Osaka, Japan; 2 Hematology, Osaka City University Graduate School of Medicine, Osaka, Japan; 3 Kansai Health Administration Center, Nippon Telegraph and Telephone West Corporation, Osaka, Japan

**Keywords:** gamma-glutamyltransferase, proteinuria, chronic kidney disease, prospective cohort study, epidemiology

## Abstract

**Background:**

Serum gamma-glutamyltransferase has been recognized as the risk factor of cardiovascular and metabolic diseases. However, the association between serum gamma-glutamyltransferase and the risk of chronic kidney disease is not well known, and no prospective studies have examined separately the relationship of serum gamma-glutamyltransferase with the risk of proteinuria versus that of low estimated glomerular filtration rate (eGFR).

**Methods:**

We prospectively followed 9,341 Japanese men who did not have low eGFR, proteinuria, or diabetes, and did not take antihypertensive medications at entry for the analysis of proteinuria, and we followed 9,299 men for the analysis of low eGFR. We defined “persistent proteinuria” as proteinuria detected two or more times consecutively and persistently as ≥1+ on urine dipstick at the annual check-up until the end of follow-up. Low eGFR was defined as eGFR <60 mL/min/1.73 m^2^.

**Results:**

During the 11-year observation period, 151 men developed persistent proteinuria and 1,276 men developed low eGFR. In multivariate models, the highest quartile (≥71 IU/L) of serum gamma-glutamyltransferase was independently related to the development of persistent proteinuria (hazard ratio 3.39; 95% confidence interval, 1.92–5.97) compared with the lowest quartile (≤25 IU/L). In joint analysis of alcohol consumption and serum gamma-glutamyltransferase, non-drinkers in the highest tertile (≥58 IU/L) of serum gamma-glutamyltransferase had the highest risk of persistent proteinuria. However, there was no association between serum gamma-glutamyltransferase and low eGFR.

**Conclusion:**

In middle-aged Japanese men, elevated serum gamma-glutamyltransferase was independently associated with future persistent proteinuria, but not with low eGFR.

## INTRODUCTION

Serum gamma-glutamyltransferase (GGT) has been widely known as a liver function test used for the assessment of liver disease and a biological marker of alcohol consumption.^1^ Previous epidemiological studies have shown that serum GGT might be a useful marker for oxidative stress.^2^ In addition, serum GGT has been reported to be related to the development of hypertension,^3^^,^^4^ stroke,^5^ and cardiovascular mortality.^6^

Chronic kidney disease (CKD) is a global health problem and has been related to the development of end-stage renal disease and cardiovascular mortality.^7^ Only three prospective cohort studies have suggested that serum GGT level was related to the development of CKD.^8^^–^^10^ Two of these studies defined CKD as a composite endpoint of the presence of an estimated glomerular filtration rate (eGFR) of <60 mL/min/1.73 m^2^ or proteinuria,^8^^,^^9^ which might lead to an attenuation of the association between serum GGT level and the risk of CKD because higher eGFR was reported to be related to the development of proteinuria.^11^ Although the other study defined CKD as eGFR <60 mL/min/1.73 m^2^, it did not examine the development of proteinuria.^10^ Only one prospective cohort study showed the relationship between serum GGT level and the incidence of microalbuminuria, but it did not take into account the influence of participants with transient albuminuria because incident microalbuminuria was measured only one time.^12^ To our knowledge, studies examining separately the effect of serum GGT level on the risk of proteinuria versus that of low eGFR in the same cohort are lacking.

GGT is a common marker for alcohol intake and correlates with alcohol intake.^13^^,^^14^ Although various prospective studies have evaluated the relationship between alcohol consumption and the risk of low eGFR and/or that of proteinuria or albuminuria,^15^^–^^25^ the results of these studies were inconclusive. Furthermore, as far as we know, there has been no prospective study that has examined the joint relationship of serum GGT and alcohol consumption to the risk of low eGFR versus that of proteinuria.

Therefore, our specific purposes were: 1) to prospectively examine separately the relationship of serum GGT level with the risk of proteinuria versus that of low eGFR and 2) to prospectively investigate the joint relationship of serum GGT and alcohol consumption with the risk of these outcomes. To take into account the influence of transient proteinuria, the definition of persistent proteinuria was used.

## MATERIALS AND METHODS

### Site and setting

The Kansai Healthcare Study is an ongoing prospective cohort study designed to obtain information on the risk factors of cardiovascular and metabolic diseases. From April 2000 to March 2001, we enrolled 12,647 male workers aged 40–55 years of a company in the Kansai area of Japan. The details of this study have been mentioned previously.^26^^,^^27^ The protocol for this research was approved by the Ethics Committee of Osaka City University Graduate School of Medicine.

The current investigation is composed of 10,019 men who had no proteinuria, an eGFR ≥60 mL/min/1.73 m^2^, a fasting plasma glucose (FPG) <126 mg/dL, and were not taking hypoglycemic agents, insulin, or antihypertensive drugs at entry. We excluded 320 men who did not undergo annual medical examinations. Because there were missing covariates at baseline, we excluded 358 men for the analysis examining the outcome of proteinuria and 400 men for that of low eGFR. Thus, a total 9,341 men remained for the analytic cohort for proteinuria and 9,299 men for low eGFR.

### Data collection and measurements

The participants were examined on a medical history, anthropometric measurements, physical examination, self-administered questionnaires on lifestyle habits, and dipstick urinalysis. Serum GGT, aspartate aminotransferase (AST), alanine aminotransferase (ALT), FPG, creatinine, total cholesterol, triglycerides, and high-density lipoprotein cholesterol were measured. Using the Friedewald formula, low-density lipoprotein cholesterol levels were calculated in participants who had triglycerides levels <400 mg/dL.^28^ Trained nurses carried out all of the measurements. Blood samples were collected after an overnight 12-hour fast. AST and ALT were measured using the International Federation of Clinical Chemistry-recommended method.^29^^,^^30^ GGT was measured using the Japanese Society of Clinical Chemistry transferable method.^31^ In general, serum creatinine was measured using an enzymatic method with a Hitachi 7350 automatic chemistry analyzer (Hitachi Ltd., Tokyo, Japan). In 1,799 participants, serum creatinine was measured using the Jaffe method. We recalibrated the Jaffe values to enzymatic values using the equation that has been described previously in detail.^24^^,^^25^ Then eGFR was calculated using the Modification of Diet in Renal Disease study equation for Japanese men as follows: eGFR = 194 × age^−0.287^ × serum creatinine^−1.094^ (mg/dL, enzymatic method).^32^ Dipstick urinalysis was performed on clean-catch, mid-stream, and random urine specimens. The results of the urine dipstick test were recorded as negative, ±, 1+, 2+, 3+, or 4+. We measured blood pressure in a sitting position at the right arm with a standard automated sphygmomanometer (BP-203RV; Omron Colin, Tokyo, Japan, and Udex-super; ELK Osaka, Japan) after approximately 5 minutes of rest. Hypertension at baseline was defined as systolic blood pressure ≥140 mm Hg or diastolic blood pressure ≥90 mm Hg.^33^ Dyslipidemia was defined as triglycerides ≥150 mg/dL, high-density lipoprotein cholesterol <40 mg/dL, low-density lipoprotein cholesterol ≥140 mg/dL, or use of oral lipid-lowering medications. Body mass index (BMI) was calculated as the weight in kilograms divided by the square of the height in meters (kg/m^2^).

Participants filled out self-administered questionnaires on the medication information and lifestyle factors, such as smoking habits, alcohol consumption, and regular leisure-time physical activity. Regarding smoking habits, participants were divided into three groups: non-smokers, past smokers, and current smokers. As for drinking habit, the questionnaire consisted of the frequency of alcohol drinking per week and the usual amount of drinking per drinking day according to Japanese standard drinks. One Japanese standard drink is equivalent to 23 g of ethanol. Daily alcohol consumption (in grams of ethanol per day) was calculated as (the amount consumed per drinking day) × (the drinking frequency per week)/7. We asked for regular leisure-time activity using the single-item questionnaire, which had three choices: rarely, sometimes, and regular (that is, at least once a week). We dichotomized participants as engaging in leisure-time physical activity at least once a week or less than once a week. The validity of this questionnaire has been reported in detail previously.^26^

### Outcomes

Proteinuria on urine dipstick was defined as ≥1+ (≥30 mg/dL)^34^ at the annual medical examination. In order to avoid the influence of participants with transient proteinuria, more definite definition of proteinuria, “persistent proteinuria”, were used: “persistent proteinuria” was defined as proteinuria observed twice or more consecutively at the annual examination and persistently until the end of follow-up. Low eGFR was defined if eGFR was less than 60 mL/min/1.73 m^2^, regardless of the presence of proteinuria.

### Statistical analyses

We used the Cox proportional hazards model to examine separately the relationship between serum GGT level and the development of two outcomes: persistent proteinuria and low eGFR. Follow-up of each participant was continued until the diagnosis of the outcome occurrence or until the 11-year follow-up examination from April 1, 2011 to March 31, 2012, whichever came first. The presence of non-linear effects of continuous independent variables was assessed by plotting the regression coefficients against the variables.^35^ We also assessed these effects using quadratic, square root, and log transformations. The proportional hazards assumption for covariates was assessed via the insertion of time-dependent covariates or using the Schoenfeld residuals plot and Schoenfeld residuals test.^36^ To test whether there was effect modification, the first-order interaction term between serum GGT and daily alcohol consumption was examined. In this study, this interaction term was not detected significantly. Multicollinearity was assessed using the variance inflation factor.^37^ We ensured lack of multicollinearity. Outliers were checked by plotting the likelihood displacement values and LMAX values of all independent variables.^36^ We did not detect outliers. To test for linear trends across quartiles of serum GGT, we modeled the median value of each quartile category as a continuous variable. We calculated the 95% confidence interval for each hazard ratio. All reported *P*-values were two-tailed. Statistical analyses were performed using Stata MP, Version 14.1 (Stata Corp., College Station, TX, USA).

## RESULTS

### Baseline characteristics

The baseline characteristics of participants according to baseline serum GGT for analysis of the development of persistent proteinuria are shown in Table 1. Participants who had higher serum GGT quartiles had higher BMI, systolic blood pressure, diastolic blood pressure, AST, ALT, total cholesterol, triglycerides, FPG, and daily alcohol consumption. They also had higher proportion of hypertension, dyslipidemia, and drinking habits and were less likely to have leisure-time physical activity. These characteristics of participants for incident low eGFR tended to be similar to those for incident persistent proteinuria (eTable 1).

**Table 1.** Baseline characteristics of study participants for incident persistent proteinuria according to quartile of baseline serum GGT

	Total	Quartile of serum GGT, IU/L			

		Quartile 1, 5–25	Quartile 2, 26–39	Quartile 3, 40–70	Quartile 4, 71–1,530
Number	9,341	2,504	2,219	2,286	2,332
Age, years	48.2 (4.2)	48.3 (4.2)	48.3 (4.2)	48.2 (4.2)	48.0 (4.1)
Body mass index, kg/m^2^	23.2 (2.8)	22.2 (2.6)	23.2 (2.7)	23.8 (2.8)	23.9 (3.0)
Systolic blood pressure, mm Hg	127.6 (17.7)	122.6 (16.5)	125.9 (17.0)	129.3 (17.2)	133.0 (18.3)
Diastolic blood pressure, mm Hg	79.7 (11.8)	76.5 (11.2)	78.3 (11.4)	80.8 (11.6)	83.2 (11.9)
Hypertension,^a^ %	28.1	18.5	24.4	31.1	39.2
AST, IU/L	22 (19–28)	19 (17–22)	21 (18–24)	24 (20–28)	28 (23–36)
ALT, IU/L	23 (17–34)	17 (14–23)	21 (16–28)	26 (19–36)	34 (25–48)
Total cholesterol, mg/dL	204.6 (33.2)	198.4 (31.1)	204.1 (31.9)	206.5 (32.5)	209.9 (35.9)
Triglycerides, mg/dL	139.7 (111.7)	105.4 (73.1)	126.3 (86.3)	143.0 (102.7)	186.0 (152.5)
High-density lipoprotein cholesterol, mg/dL	56.5 (14.8)	56.0 (14.1)	55.8 (14.8)	56.1 (14.9)	58.2 (15.5)
Low-density lipoprotein cholesterol,^b^ mg/dL	121.5 (31.1)	121.6 (28.9)	123.5 (30.0)	122.7 (30.8)	118.2 (34.2)
Dyslipidemia,^b,c^ %	50.4	39.7	48.1	52.8	61.8
Fasting plasma glucose, mg/dL	97.2 (9.2)	95.1 (8.8)	96.9 (8.8)	98.1 (9.2)	98.9 (9.6)
Estimated glomerular filtration rate, mL/min/1.73 m^2^	84.8 (14.1)	84.8 (14.4)	84.1 (13.9)	84.2 (13.8)	86.2 (14.1)
Daily alcohol consumption,^d^ g ethanol/day	23.0 (3.3–46.0)	8.2 (0–23.0)	16.4 (3.3–32.9)	23.0 (14.8–46.0)	41.1 (23.0–46.0)
Drinking habit, %	85.0	72.0	83.3	90.1	95.7
Smoking habit					
Non-smokers, %	21.2	24.0	22.2	21.0	17.3
Past smokers, %	21.6	21.5	20.0	24.2	20.7
Current smokers, %	57.2	54.5	57.8	54.8	62.0
Regular leisure-time physical activity, %	17.9	19.4	18.7	18.2	15.3

ALT, alanine aminotransferase; AST, aspartate aminotransferase; GGT, gamma-glutamyltransferase.

Note: Values are the mean (SD), the median (interquartile range) or %.

^a^Hypertension: Systolic blood pressure ≥140 mm Hg or diastolic blood pressure ≥90 mm Hg.

^b^Low-density lipoprotein cholesterol levels were calculated by the Friedewald formula for participants with triglyceride levels <400 mg/dL. We excluded 230 participants with triglyceride levels ≥400 mg/dL.

^c^Dyslipidemia was defined as triglycerides ≥150 mg/dL, high-density lipoprotein cholesterol <40 mg/dL, low-density lipoprotein cholesterol ≥140 mg/dL, or use of oral lipid-lowering medications.

^d^Daily alcohol consumption: (the amount consumed per drinking day) × (the drinking frequency per week)/7.

### Prospective analysis

During the total follow-up of 84,587 person-years, 151 incident cases of persistent proteinuria were observed. Incidence rates and multivariate-adjusted hazards ratios are shown in Table 2. In Cox proportional hazards models, as relationships of BMI and daily alcohol consumption with the development of persistent proteinuria were nonlinear, we fitted models using these categorized variables. Therefore, the BMI was divided into the following four categories: BMI <18.5, 18.5 to <25.0, 25.0 to <30.0, and ≥30.0 kg/m^2^, according to the World Health Organization classification of obesity.^38^ As for drinking habits, except non-drinkers, participants were classified into tertiles of daily alcohol consumption levels. After adjustment for age, BMI categories, FPG, smoking habits, regular leisure-time activity, and hypertension, elevated serum GGT was related to the development of persistent proteinuria. In addition, even after adjustment for daily alcohol consumption, the results did not change. Compared to non-drinkers, participants who drank 16.5–42.7 g ethanol per day had the lowest risk of the development of persistent proteinuria (model 2 of Table 2). After further adjustment for baseline eGFR (model 3 of Table 2) or dyslipidemia (yes/no) (data not shown), these associations did not change.

**Table 2.** Multivariate model of the incidence of persistent proteinuria according to baseline serum GGT and alcohol consumption (*n* = 9,341)

	Incidence rates,^a^ (cases/person-years)	Multiple-adjusted hazard ratio (95% CI)		

		Model 1^b^	Model 2^c^	Model 3^d^
Serum GGT, IU/L				
Quartile 1, 5–25	0.82 (19/23,249)	1.00 (reference)	1.00 (reference)	1.00 (reference)
Quartile 2, 26–39	1.65 (33/19,990)	1.73 (0.98–3.06)	1.88 (1.06–3.34)	1.89 (1.07–3.35)
Quartile 3, 40–70	1.65 (34/20,638)	1.58 (0.89–2.79)	1.85 (1.03–3.31)	1.85 (1.03–3.33)
Quartile 4, 71–1,530	3.14 (65/20,710)	2.74 (1.62–4.63)	3.44 (1.95–6.05)	3.39 (1.92–5.97)
*P* for trend		<0.001	<0.001	<0.001
Daily alcohol consumption				
Non-drinkers	1.92 (24/12,470)		1.00 (reference)	1.00 (reference)
0.1–16.4 g ethanol/day	1.75 (47/26,849)		0.80 (0.49–1.32)	0.80 (0.49–1.32)
16.5–42.7 g ethanol/day	1.49 (35/23,528)		0.52 (0.30–0.89)	0.50 (0.29–0.87)
≥42.8 g ethanol/day	2.07 (45/21,740)		0.59 (0.34–1.02)	0.58 (0.34–1.01)

CI, confidence interval; GGT, gamma-glutamyltransferase.

^a^Incidence rates are expressed as the incidence per 1,000 person-years.

^b^Model 1 was adjusted for age, body mass index (<18.5, 18.5–24.9, 25.0–29.9, ≥30.0), fasting plasma glucose, smoking habits (nonsmokers, past smokers and current smokers), regular leisure-time physical activity (yes/no), and hypertension (yes/no).

^c^Model 2 was adjusted for all variables in model 1 of Table 2 plus daily alcohol consumption (nondrinkers, 0.1–16.4, 16.5–42.7, ≥42.8 g ethanol/day).

^d^Model 3 was adjusted for all variables in model 2 of Table 2 plus estimated glomerular filtration rate.

During the total follow-up of 80,824 person-years, 1,276 incident cases of low eGFR were observed. Incidence rates and multivariate-adjusted hazards ratios are shown in Table 3. In Cox proportional hazards models, as relationships of BMI, FPG, daily alcohol consumption, and eGFR with the development of low eGFR were nonlinear, we fitted a model using these categorized variables. The manner of categorization of BMI and daily alcohol consumption was similar to that of Table 2. The FPG level was classified into the following two categories: FPG <100 and FPG ≥100 mg/dL in the model of Table 3. As for baseline eGFR, we divided the eGFR level into tertiles. In multivariable models of Table 3, elevated serum GGT was not associated with the incident low eGFR. On the other hand, participants who drank higher daily alcohol had a lower risk of the development of low eGFR compared with non-drinkers in the models that included serum GGT and daily alcohol consumption simultaneously (models 2 and 3 of Table 3).

**Table 3.** Multivariate model of the incidence of low eGFR according to baseline serum GGT and alcohol consumption (*n* = 9,299)

	Incidence rates,^a^ (cases/person-years)	Multiple-adjusted hazard ratio (95% CI)		

		Model 1^b^	Model 2^c^	Model 3^d^
Serum GGT, IU/L				
Quartile 1, 5–25	16.4 (361/22,052)	1.00 (reference)	1.00 (reference)	1.00 (reference)
Quartile 2, 26–39	16.8 (319/19,005)	1.02 (0.87–1.19)	1.07 (0.92–1.25)	1.05 (0.90–1.22)
Quartile 3, 40–70	15.6 (307/19,726)	0.92 (0.79–1.07)	1.01 (0.86–1.18)	0.97 (0.83–1.15)
Quartile 4, 71–1,530	14.4 (289/20,041)	0.89 (0.76–1.05)	1.03 (0.86–1.22)	1.08 (0.91–1.29)
*P* for trend		0.1	0.9	0.4
Daily alcohol consumption				
Non-drinkers	19.3 (228/11,838)		1.00 (reference)	1.00 (reference)
0.1–16.4 g ethanol/day	17.5 (445/25,436)		0.92 (0.78–1.08)	0.96 (0.81–1.12)
16.5–42.7 g ethanol/day	14.5 (326/22,489)		0.74 (0.62–0.89)	0.84 (0.70–1.00)
≥42.8 g ethanol/day	13.2 (277/21,061)		0.70 (0.58–0.84)	0.77 (0.64–0.93)

CI, confidence interval; eGFR, estimated glomerular filtration rate; GGT, gamma-glutamyltransferase.

^a^Incidence rates are expressed as the incidence per 1,000 person-years.

^b^Model 1 was adjusted for age, body mass index (<18.5, 18.5–24.9, 25.0–29.9, ≥30.0), fasting plasma glucose (<100, ≥100 mg/dL), smoking habits (nonsmokers, past smokers and current smokers), regular leisure-time physical activity (yes/no), and hypertension (yes/no).

^c^Model 2 was adjusted for all variables in model 1 of Table 3 plus daily alcohol consumption (nondrinkers, 0.1–16.4, 16.5–42.7, ≥42.8 g ethanol/day).

^d^Model 3 was adjusted for all variables in model 2 of Table 3 plus eGFR (<79.2, 79.3–90.7, ≥90.8 mL/min/1.73 m^2^).

For sensitivity analysis, we excluded participants who had the upper 2.5th percentile value of GGT or participants who had systolic blood pressure ≥140 mm Hg or diastolic blood pressure ≥90 mm Hg in the baseline population. However, the results of examining separately the relationship of serum GGT with the development of persistent proteinuria and low eGFR did not change (data not shown). To avoid the influence of participants with liver dysfunction, we excluded participants with ALT ≥40 IU/L. After excluding 1,595 of the 9,341 participants in the analytic cohort for proteinuria, the results did not change (data not shown).

**Figure 1.**
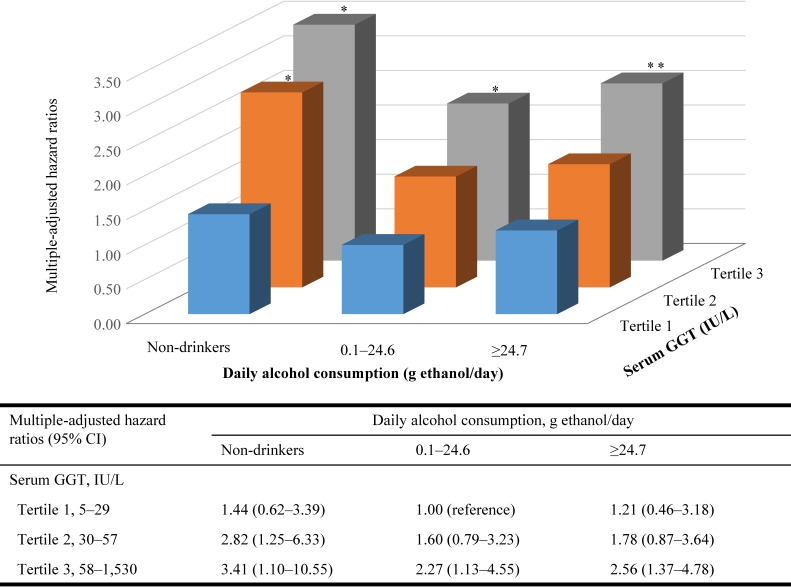
Multiple-adjusted hazard ratios for persistent proteinuria according to joint categories of daily alcohol consumption and serum GGT. Multiple-adjusted hazard ratios were calculated after adjustment for age, body mass index (<18.5, 18.5–24.9, 25.0–29.9, ≥30.0), fasting plasma glucose, smoking habits (nonsmokers, past smokers and current smokers), regular leisure-time activity (yes/no), and hypertension (yes/no). ^*^: *P* < 0.05, ^**^: *P* < 0.01. CI, confidence interval; GGT, gamma-glutamyltransferase.

The joint analysis of daily alcohol consumption and serum GGT in relation to the development of persistent proteinuria is shown in Figure 1. Using too many categories might cause too low incidence in each category. Therefore, apart from non-drinkers, we divided participants into two groups by median of daily alcohol consumption levels. In the joint analysis, participants who drank 0.1–24.6 g ethanol per day and had the lowest tertile of serum GGT had the lowest risk of the development of persistent proteinuria. Compared with this category, non-drinkers who had the highest tertile of serum GGT had the highest risk of the development of persistent proteinuria. In each GGT category, the relationship between daily alcohol consumption and the development of persistent proteinuria tended to be U-shaped. On the other hand, in each daily alcohol consumption category, we observed a dose-response relationship between serum GGT and the development of persistent proteinuria.

## DISCUSSION

These prospective data demonstrated that serum GGT was independently related to the development of persistent proteinuria. This association was independent of age, BMI categories, FPG, smoking habits, regular leisure-time physical activity, hypertension, daily alcohol consumption, and eGFR. According to joint categories of daily alcohol consumption and serum GGT, non-drinkers who had the highest tertile of serum GGT had the highest risk of the development of persistent proteinuria, but participants who drank 0.1–24.6 g ethanol per day and had the lowest tertile of serum GGT had the lowest risk. In contrast, no association was found between elevated serum GGT and the risk of low eGFR.

Three previous prospective cohort studies reported the association between serum GGT and the development of CKD.^8^^–^^10^ Ryu et al^8^ reported in 10,337 Korean male workers that elevated serum GGT was related to the development of CKD, but the multivariate models were not adjusted for alcohol consumption. Shen et al^9^ showed in 21,818 urban Han Chinese that the presence or absence of alcohol drinking was not related to the development of CKD and that men, but not women, with elevated serum GGT had an increased risk of CKD. Both studies^8^^,^^9^ defined CKD using a composite endpoint, which was defined as the presence of an eGFR of <60 mL/min/1.73 m^2^ or proteinuria. The relationships of serum GGT level with the risk of proteinuria versus that of low eGFR have not previously been examined separately. Kunutsor et al^10^ showed in 2,338 Finnish men that the relationship between serum GGT and the risk of CKD, defined as an eGFR of <60 mL/min/1.73 m^2^, was a log-linear association in age-adjusted analysis. However, the association did not remain statistically significant after further adjustment, including for alcohol consumption. In their study, participants with an eGFR <60 mL/min/1.73 m^2^ at baseline were excluded but they did not consider the presence or absence of proteinuria. Lee et al^12^ reported in 2,478 black and white men and women that the relationship between serum GGT and incident microalbuminuria was a U-shaped association in nonhypertensive and nondiabetic participants in multivariate models that included alcohol consumption. As incident microalbuminuria was measured only one time, they did not avoid the influence of participants with transient albuminuria. Furthermore, their inconsistent results with ours might be in part due to differences in ethnicity, age distribution, and GGT distribution of study subjects.^1^

On the other hand, a number of prospective studies have assessed the relationship between alcohol consumption and the risk of the development of CKD.^15^^–^^25^ Regarding the development of low eGFR, four studies have reported that alcohol consumption was not related to the risk of low eGFR or eGFR decline speed,^15^^–^^18^ whereas six studies have shown that alcohol consumption reduced the risk of low eGFR.^19^^–^^24^ As for the development of proteinuria or albuminuria, four prospective studies have reported the association between alcohol consumption and the incidence of proteinuria or albuminuria.^20^^,^^22^^,^^23^^,^^25^ Alcohol consumption was related to a reduced risk of the development of proteinuria or albuminuria in three studies,^22^^,^^23^^,^^25^ but it was related to an increased risk of the development of albuminuria in one study.^20^ In these studies, the multiple-adjusted models were not adjusted for serum GGT, and the joint relationship of alcohol consumption and serum GGT to the development of low eGFR or proteinuria was not examined.

In the present study, we were unable to identify why elevated serum GGT was related to the development of persistent proteinuria but not to low eGFR. The association between elevated serum GGT and incident persistent proteinuria did not change after adjustment for alcohol consumption. Serum GGT is a biological marker of alcohol intake,^1^ and we previously reported that the relationship between daily alcohol consumption and the development of proteinuria tended to be a U-shaped association.^25^ Therefore, we investigated the relationship of daily alcohol consumption and serum GGT to the development of persistent proteinuria using the joint categories. In the joint analysis, the relationship of daily alcohol consumption to the development of persistent proteinuria was a U-shaped association in each serum GGT category. Furthermore, even for non-drinkers, serum GGT was independently related to the development of persistent proteinuria in a dose-dependent manner. Several epidemiological studies have shown that serum GGT level within its normal range might be an early and sensitive enzyme associated with oxidative stress.^2^ Although it is known that reactive oxygen species play an essential role in the pathogenesis of proteinuria in the case of diabetic nephropathy, an experimental study has shown that reactive oxygen species may also cause proteinuria in various clinical conditions related to high oxidative stress.^39^ The oxidant stress might have an important role of the relationship between serum GGT and the development of persistent proteinuria.

A notable strength of our study was that we used the definition of persistent proteinuria to take into account the influence of participants who had transient proteinuria. In previous studies about the relationship of serum GGT to the development of CKD^8^^,^^9^ or albuminuria,^12^ urine samples have been collected only one time.

There were some limitations in our study. First, because the study participants were middle-aged Japanese men who worked at the same company, our results might not be generalizable to other populations. Second, as we measured proteinuria using a urinary dipstick test, the participants with incident proteinuria might include those with false-positive proteinuria and miss those with false-negative proteinuria. However, urinary dipstick testing is very convenient and easily available for clinical practice and large epidemiological studies. Additionally, the definition of persistent proteinuria was adopted to avoid overestimating false-positive proteinuria as far as possible.

In conclusion, higher serum GGT level was positively related to the incidence of persistent proteinuria but not to low eGFR in middle-aged Japanese men, independent of daily alcohol consumption. In joint analysis of serum GGT and alcohol consumption, non-drinkers who had the highest tertile of serum GGT had the highest risk of persistent proteinuria, and participants who drank 0.1–24.6 g ethanol per day and who had the lowest tertile of serum GGT had the lowest risk of development of persistent proteinuria. Further research to identify why non-drinkers with an elevated serum GGT had the higher risk of persistent proteinuria is necessary.

## APPENDIX A. SUPPLEMENTARY DATA

eTable 1. Baseline characteristics of study participants for incident low eGFR according to quartile of baseline serum GGT
