# Supplementary material for: Serum Gamma-Glutamyltransferase, Daily Alcohol Consumption, and the Risk of Chronic Kidney Disease: The Kansai Healthcare Study
Source: J Epidemiol. 2020 Apr 5;30(4):163–9. doi: 10.2188/jea.JE20180240 (PMC7064553; doi:10.2188/jea.JE20180240)
Supplement: Supplementary file 1 [file je-30-163-s001.pdf]

eTable 1. Baseline characteristics of study participants for incident low eGFR according to quartile of baseline serum GGT

	Total	Quartile of serum GGT, IU/L			
		Quartile 1, 5–25	Quartile 2, 26–39	Quartile 3, 40–70	Quartile 4, 71–1,530
Number	9,299	2,498	2,205	2,276	2,320
Age, years	48.2 (4.2)	48.3 (4.2)	48.3 (4.2)	48.2 (4.2)	48.0 (4.1)
Body mass index, kg/m^2	23.2 (2.8)	22.2 (2.6)	23.2 (2.8)	23.7 (2.8)	23.9 (3.0)
Systolic blood pressure, mm Hg	127.6 (17.7)	122.6 (16.4)	125.9 (17.0)	129.3 (17.2)	133.0 (18.3)
Diastolic blood pressure, mm Hg	79.7 (11.8)	76.5 (11.2)	78.4 (11.4)	80.8 (11.6)	83.2 (11.9)
Hypertension,[a] %	28.2	18.5	24.4	31.2	39.2
AST, IU/L	22 (19–28)	19 (17–22)	21 (18–24)	24 (20–28)	28 (23–36)
ALT, IU/L	23 (17–34)	17 (14–23)	21 (16–28)	26 (19–36)	34 (25–48)
Total cholesterol, mg/dL	204.6 (33.2)	198.3 (31.1)	204.1 (31.9)	206.5 (32.5)	209.9 (35.9)
Triglycerides, mg/dL	139.7 (111.9)	105.4 (73.1)	126.4 (86.3)	142.9 (102.8)	186.2 (152.8)
High-density lipoprotein cholesterol, mg/dL	56.5 (14.8)	56.0 (14.1)	55.8 (14.8)	56.1 (14.9)	58.2 (15.5)
Low-density lipoprotein cholesterol,[b] mg/dL	121.5 (31.0)	121.6 (28.9)	123.6 (30.0)	122.7 (30.9)	118.2 (34.2)
Dyslipidemia,[b, c] %	50.4	39.7	48.1	52.9	61.8
Fasting plasma glucose, mg/dL	97.2 (9.2)	95.1 (8.8)	96.8 (8.8)	98.1 (9.2)	98.9 (9.6)
eGFR, mL/min/1.73 m^2	84.8 (14.1)	84.8 (14.4)	84.1 (13.9)	84.2 (13.8)	86.2 (14.1)
Daily alcohol consumption,[d] g ethanol/day	23.0 (3.3–46.0)	8.2 (0–23.0)	16.4 (3.3–32.9)	23.0 (14.8–46.0)	41.1 (23.0–46.0)
Drinking habit, %	85.1	72.1	83.4	90.1	95.7
Smoking habit					
Non-smokers, %	21.1	24.0	22.1	21.0	17.2
Past smokers, %	21.6	21.5	20.0	24.2	20.6

Current smokers, %	57.3	54.5	57.9	54.8	62.2
Regular leisure-time physical activity, %	17.9	19.4	18.6	18.2	15.4

ALT, alanine aminotransferase; AST, aspartate aminotransferase; eGFR, estimated glomerular filtration rate; GGT, gamma-glutamyltransferase.

Note: Values are the mean (SD), the median (interquartile range) or the number (%).

[a]Hypertension: Systolic blood pressure ≥140 mm Hg or diastolic blood pressure ≥90 mm Hg.

[b]Low-density lipoprotein cholesterol levels were calculated by the Friedewald formula for subjects with triglyceride levels <400 mg/dL. We excluded 230 subjects with triglyceride levels ≥400 mg/dL.

[c]Dyslipidemia was defined as triglycerides ≥150 mg/dL, high-density lipoprotein cholesterol <40 mg/dL, low-density lipoprotein cholesterol ≥140 mg/dL, or use of oral lipid-lowering medications.

[d]Daily alcohol consumption: (the amount consumed per drinking day) × (the drinking frequency per week)/7.
